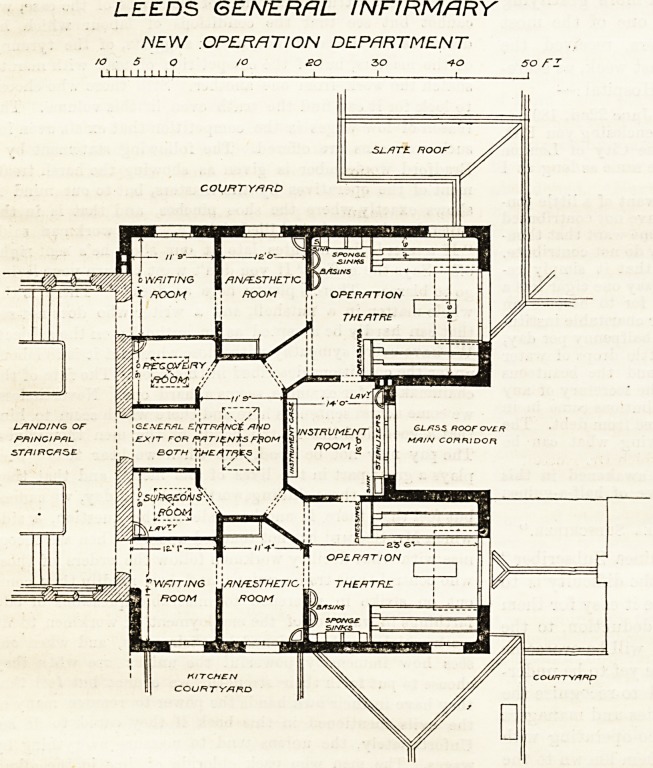# Hospital Construction

**Published:** 1897-07-03

**Authors:** 


### The Institutional Workshop.

### HOSPITAL CONSTRUCTION.

### LEEDS GENERAL INFIRMARY.—NEW OPERATING DEPARTMENT.

The operating department of this institution is being remodelled, as shown on the accompanying plan, in which the north point is at the top. The new building is on an upper floor, so that a good top light will be secured to the two theatres. Each theatre has a waiting-room and anæsthetic-room attached, and the instrument-room lies

LEEDS GENERAL INFIRMARY
NEW OPERATION DEPARTMENT

between the theatres (with access from both), the general arrangement being suitable and convenient. The two rooms allotted to the surgeon and to recovery, however, are lighted only by skylights, and this, like the similar lighting of the central lobby, might apparently have been avoided by a modified plan, for direct light and ventilation by windows is very desirable for such places. The two theatres and the instrument-room will have floors of terrazzo and wall-linings of opaline, a glass tile which is likely, from its beautiful surface, to come into favour for hospital work; but it must not be exposed to rough usage, as it fractures much more easily than the ordinary glazed tiles or bricks. It is stated that the windows will be "double-glazed" with plate glass; but this system, unless provision is made for access to the space between the plates, is not satisfactory, and has been abandoned elsewhere after trial. The new building will be lighted by electricity, and the heating and ventilation will be on the system adopted by Mr. Key in other institutions, in which air (heated when required), is forced by fans into the rooms, through inlets at the ceiling level, and finds its exit by outlets near the floor. We have on other occasions expressed our opinion on the doubtful gain of using mechanical ventilation for hospital work, our experience being that it is never a necessity, and that unless it is kept in perfect order day by day, by an expert who thoroughly understands it, the result it certain in time to be worse than if the building had been planned for natural ventilation alone.

## Figures and Tables

**Figure f1:**